# Cattle welfare assessment based on adaptive fuzzy logic and multimodal data fusion

**DOI:** 10.3389/fvets.2025.1568715

**Published:** 2025-04-09

**Authors:** Lei Tong, Jiandong Fang, Xiuling Wang, Yudong Zhao

**Affiliations:** ^1^College of Information Engineering, Inner Mongolia University of Technology, Hohhot, China; ^2^Inner Mongolia Key Laboratory of Intelligent Perception and System Engineering, Hohhot, China; ^3^Inner Mongolia Synergy Innovation Center of Perception Technology in Intelligent Agriculture and Animal Husbandry, Hohhot, China

**Keywords:** intelligent ranch, welfare evaluation, multimodal data, cattle behavior recognition, fuzzy neural network

## Abstract

This study proposes a cattle welfare evaluation method based on multi-modal data fusion, which integrates various data dimensions, such as cattle behavior characteristics, feeding management conditions, and environmental parameters, to achieve a systematic assessment of cattle welfare levels. The method establishes a quantitative scoring system based on behavioral duration and individual group differences, and designs a multi-modal data processing framework that combines Backpropagation (BP) neural networks with adaptive fuzzy logic. This framework uses a Gaussian membership function to replace the traditional triangular membership function for feature mapping, significantly improving the robustness and accuracy of the evaluation system through a differentiated weight allocation strategy. By introducing a dynamic adaptive scoring mechanism, the model can automatically adjust evaluation parameters according to the actual application scenario, ensuring the practicality and reliability of the evaluation results. Experimental validation shows that the method performs excellently across the three evaluation dimensions of environment, feeding, and behavior: the environment evaluation module achieves accuracy rates of 88.7% and 95.0% for the training and validation sets, respectively; the feeding evaluation module achieves 98.3% and 100%, respectively; and the behavior evaluation module achieves 85.7% and 93.6%. The validation accuracy for all dimensions exceeds 90%. This method integrates multi-modal data, providing a reliable decision support tool for modern farms. It demonstrates strong adaptability and can be adjusted to suit different environments. The research results are of significant importance for promoting the intelligent transformation of farm management, contributing to enhancing operational efficiency and sustainability in farms of varying types and scales.

## 1 Introduction

Animal welfare, as a core element of sustainable livestock production, is increasingly becoming a global focal point (1, 2). In particular, in dairy cattle farming, good welfare conditions not only directly impact production efficiency and product quality, but also reflect the ethical standards of modern animal husbandry. To ensure that welfare standards are met, the industry has gradually adopted integrated evaluation systems, which objectively assess multiple indicators, including individual animal performance, environmental resource allocation, and management practices (3). Concern for animal welfare is not only a moral responsibility but also a sound business decision beneficial to all stakeholders (4). Therefore, scientifically and systematically evaluating cattle welfare has become a key focus in livestock research and management.

With the growing public concern for animal welfare, an increasing number of studies have explored how to assess animals’ health and welfare levels by monitoring and analyzing their natural behaviors (5, 6). Cattle behavior, as a direct indicator of their health, is crucial for enhancing farm management practices. Monitoring these behaviors not only helps managers improve livestock management but also provides data for informed decision-making, ultimately leading to the implementation of strategies to improve cattle welfare (7). Currently, research on cattle welfare assessment primarily focuses on unidimensional data analysis. In the area of behavior recognition, Tun et al. (8) proposed a more accurate, real-time lameness detection method, enabling farmers to promptly identify and address lameness issues, providing precise references for cattle health management and early intervention. Gao et al. (9) developed a method for recognizing cattle rumination behavior based on the extraction of maxillofacial skeletal features, which not only supports the automation of rumination behavior recognition but also provides accurate references for evaluating cattle health. Wang et al. (10) employed a dynamic small-area tracking discrimination algorithm to assess cattle emotional states through ear movement analysis, advancing emotional monitoring technology and helping to better understand cattle mental health. Shloma et al. (11) utilized convolutional neural networks for recognizing cattle feeding behavior. This study is significant as precise monitoring of feeding behavior effectively evaluates cattle nutrition and welfare levels. However, these methods are often limited to the analysis of individual behavioral features and struggle to provide a comprehensive reflection of cattle welfare. Therefore, integrating multidimensional behavioral data for a holistic assessment of cattle welfare remains a critical direction for future research.

As the link between farm animal welfare and environmental sustainability becomes increasingly important, there is a growing need for innovative solutions to protect animal welfare (12). The improvement of farm animal welfare not only depends on the animals’ own behaviors but also closely ties to management practices, particularly the optimization of environmental conditions and feeding management, which play a crucial role in enhancing animal health and welfare. In cattle welfare assessment, environmental conditions and feeding management are also key factors (13). Effective environmental management reduces disease transmission and stress responses, improving cattle comfort. Research by Yangchun et al. (14) demonstrates that cattle are highly sensitive to environmental changes, and unsuitable conditions can decrease production performance and disease resistance, significantly impacting economic outcomes. Bakony et al. (15) focused solely on surface body temperature as a heat stress evaluation indicator, while Kim et al. (16) proposed a simplified Animal Welfare Assessment Grid (S-AWAG), but the evaluation dimensions are relatively limited. In feeding management research, Ferraz et al. (17) only compared the effects of different bedding materials on dairy cattle comfort. These unimodal research approaches struggle to comprehensively reflect the multidimensional nature of cattle welfare, and the accuracy and reliability of evaluation results need further improvement.

To address the limitations of unidimensional assessment methods in existing research, this study proposes a cattle welfare evaluation method based on adaptive fuzzy logic and multimodal data fusion. The method is innovative in several aspects: first, it constructs a comprehensive evaluation system encompassing three dimensions—environment, feeding, and behavior—effectively representing multidimensional data by quantifying behavioral duration and individual differences within the herd; second, it employs three parallel Backpropagation (BP) neural networks for feature extraction and preliminary classification, incorporating a Gaussian membership function to enhance system robustness; and finally, it introduces a differentiated weight distribution mechanism and a dynamic adaptive scoring mechanism, improving the system’s ability to identify key states. Supported by big data technology, the proposed method effectively integrates multi-source heterogeneous data (18), providing farm managers with more comprehensive and accurate decision-making support.

In conclusion, the integration of multisource data—combining cattle behavior, environmental conditions, and feeding management—enables a more comprehensive and accurate assessment of cattle health and welfare. This fusion approach effectively consolidates data from diverse sources, assisting farm managers in making informed decisions that enhance cattle welfare, production efficiency, and farm profitability. The application of multisource data fusion technology in livestock management holds promising prospects and contributes to the advancement of modern livestock farming toward intelligence and sustainability.

## 2 Materials and methods

### 2.1 Materials

#### 2.1.1 Behavioral data

Cattle behavior data is a crucial basis for assessing welfare status. By monitoring various behaviors, it provides a comprehensive reflection of cattle adaptability and health in their rearing environment (19). According to the internationally recognized animal welfare evaluation guideline, the “5F” principle, behaviors are categorized into three groups: physiological, locomotor, and social behaviors (20, 21). These behavior data not only assess the current living conditions of cattle but also reveal potential health risks. To present the classification and characteristics of behavior data more intuitively, this study refines the behavior categories based on the “5F” principle and provides detailed descriptions of the classifications, characteristics, and perception methods in Table 1.

**Table 1:** Behavior data.

No.	Behavior classification	Specific behavior	Behavior characteristics	Perception methods
1	Physiological behavior	Feeding	Duration, frequency and interval of feeding. Under normal circumstances, feeding time is within a certain range, and eating too short or too long may reflect feed quality problems or health problems.	Visual perception
2	Physiological behavior	Drinking	The frequency, duration and amount of drinking water. Abnormal drinking behavior, such as too high or too low frequency of drinking water, may indicate abnormal ambient temperature or cattle health problems (such as fever, stress).	Visual perception
3	Physiological behavior	Standing	Time, frequency and duration of standing. Long-term standing or frequent standing may mean uncomfortable environment, pain or unsuitable padding.	Visual perception
4	Physiological behavior	Lying	The length of lying time, posture and number of ups and downs. Adequate lying time is very important for the rumination and comfort of cattle. Too much or too little lying may indicate health problems (such as leg pain and environmental discomfort).	Visual perception
5	Sports behavior	Walking	The number of walks, steps, distance. Normal walking can promote health, but insufficient walking may indicate laziness or leg problems, and excessive walking may be due to anxiety or environmental discomfort.	Visual perception
6	Social behavior	Fighting	Including the frequency and intensity of the fight between cattle. Frequent fighting may be a manifestation of social pressure or lack of space.	Visual perception
7	Social behavior	Climbing	This behavior is generally related to estrus. If the non-estrous climbing behavior is frequent, it may indicate group management problems or excessive pressure on cattle.	Visual perception

The behavioral data were obtained through continuous recording by surveillance cameras installed inside the dairy barn. The cameras used were from the Hikvision brand, with a video resolution of 1,920 × 1,080 pixels and a frame rate of 25 frames per second. The subjects of the recordings were lactating Holstein cattle (approximately 34–36 months old), located in the cattle pen on one side of the barn. Due to the fixed position of the cameras in the middle section of the barn, only one-half of the cattle pen on one side was captured, monitoring approximately 20–25 cattle. To enable efficient monitoring and analysis of cattle behavior, the surveillance system recorded the daily activities of the cattle and converted them into video clips. Given the limited processing power of edge devices, and to ensure efficient data transmission and real-time processing, the full-day video was divided into 10-min segments, generating a total of 43 clips. Considering that cattle move slowly and the duration of their behavior is unpredictable, we selected 43 representative video clips from the day’s footage as the source for behavioral data analysis. The remaining 101 clips were included in the dataset for cattle behavior recognition and tracking. The segmentation of the video not only alleviated the burden on edge devices but also improved the flexibility and accuracy of the analysis.

Using YOLO-based object detection techniques along with object tracking technologies such as ByteTrack and DeepSORT, cattle behavior in the video is identified and analyzed. This includes extracting physiological behaviors such as feeding, drinking, standing, and lying down, as well as movement and social behaviors like walking, fighting, and climbing. The duration and frequency of these behaviors are recorded. Object detection is used to accurately locate the cattle within the video, while object tracking ensures continuous monitoring and recording of individual cattle behaviors. The identification results for each ID and its behaviors are stored in text files, including the category and duration of each behavior, providing reliable data for subsequent scoring and decision-making.

Based on the acquisition and analysis of behavioral data, this study proposes a method for evaluating cattle welfare. By dynamically analyzing and classifying the distribution of group and individual behaviors, this method effectively reflects the welfare level of cattle under different environmental conditions. The main steps of the welfare evaluation are outlined below:

(1) Setting the behavior baseline for time periods: The behavior baseline for different time periods is dynamically established based on the cattle’s biological rhythms and behavioral characteristics. For example:

- Daytime (06:00–18:00): Feeding and walking activities increase, with the proportion of feeding activities reaching 30%–50%.
- Evening (18:00–22:00): During this period, cattle typically reduce their activity levels and enter a more static state. The proportion of feeding decreases, while the proportion of lying down should reach 40%–60%.
- Nighttime (22:00–06:00): Most cattle should be in a lying down state, with the proportion of lying down reaching 60%–80%.

The behavior baseline is dynamically adjusted based on historical data and group behavior patterns. If the behavior pattern within a given time period significantly deviates from the baseline, it indicates a potential anomaly during that period.

(2) Group behavior pattern analysis: By analyzing the behavioral data of the majority of cattle in the group, the prevailing behavior patterns during the monitoring period are identified. For example:

- If 70% of the cattle are in a lying state during a given time period, the prevailing behavior for that period can be identified as lying.

Group behavior pattern analysis provides an important reference baseline for individual behavior assessment. If a cattle’s behavior significantly deviates from the prevailing group pattern (e.g., when most cattle are lying, and the cattle remains standing), it may indicate potential health or welfare issues.

(3) Individual behavior duration analysis: In-depth analysis of each cattle’s behavioral data is conducted to calculate the duration and proportion of each behavior category. For example:

- The total monitoring time for Cattle A was 10 min, during which the cow was lying down for 5 min (50%), walking for 30 s (5%), feeding for 3 min (30%), and standing for 1 min and 30 s (15%).

By comparing individual behavior with the group baseline, a more accurate assessment of the rationality of the individual behavior can be made. If a cattle’s lying down time is significantly lower than the group baseline (e.g., the group baseline is 70%, but the cattle’s is only 40%), it may indicate a potential issue.

(4) Welfare scoring criteria and rule development: Based on the differences between individual and group behaviors, and in combination with the behavior baseline for specific time periods, welfare scoring standards are established. For example:

- If the individual behavior meets the benchmark and is consistent with the mainstream model of the group, a higher score can be given;
- If the individual behavior deviates from the benchmark or is quite different from the mainstream model of the group, the score is reduced accordingly.

According to the difference between individual behavior and group behavior, the welfare scoring standard is set according to the time period benchmark. The scoring rules start from the following three dimensions:

Behavior rationality score: This score assesses whether an individual’s actions align with the baseline for a specific time period, with the calculation method described in Equation (1).

(1) $$S_{R}=1-|b_{g}-Q|$$

Where $b_{g}$ is the proportion of individual behavior, and Q is the group benchmark.

Group consistency score: This score evaluates the alignment between an individual’s actions and the prevailing behaviors of the group, with the calculation formula provided in Equation (2).

(2) $$S_{G}=1-|b_{g}-b_{q}|$$

Where $b_{q}$ is the proportion of group mainstream behavior.

Persistent deduction: For some abnormal behaviors (such as continuous standing or repeated feeding), the deduction rules are set according to their duration, with the calculation formula provided in Equation (3).

(3) $$S_{D}=k\cdot M$$

Where k is the deduction coefficient, M is the duration of abnormal behavior, and the longer the duration, the more the deduction.

The final welfare score is calculated using the formula provided in Equation (4):

(4) $$S_{F}=\left(S_{R}\cdot \omega_{1}\right)+\left(S_{G}\cdot \omega_{2}\right)-S_{D}$$

Among them, $\omega_{1}$ and $\omega_{2}$ are weight parameters, which can be dynamically adjusted according to the time period.

After conducting a comprehensive evaluation of the cattle’s behavior, the welfare status of each cattle is categorized into different levels to provide a clearer reflection of the evaluation results and facilitate further analysis. This classification helps quickly identify individuals with good welfare performance or potential issues, thereby providing intuitive data support for optimizing management practices.

Behavior grading standard: According to the frequency, duration or intensity of behavior, it is divided into three grades: excellent, medium and poor.

- Excellent cattle behavior is characterized by: deviations in feeding and drinking behaviors from the group baseline within ±10%, with the time distribution in line with biological rhythms, where the daytime feeding proportion remains between 30 and 50%; balanced standing and lying behaviors, with nighttime lying time accounting for 60 to 80%, and consistency with the group’s main behavior pattern scoring no less than 0.8; deviations in activity level from the group average within ±15%; the frequency of mounting behaviors during estrus not exceeding 1.2 times the group average, with no abnormal mounting during non-estrus periods; the frequency of aggressive behavior being less than 0.5 times the group average, with each incident lasting no more than 2 min.
- Moderate cattle behavior is characterized by: deviations in feeding or drinking behaviors from the group baseline within ±10% to 25%; standing time exceeding 1.3 times the group average, or nighttime lying time accounting for 45% to 60%; deviations in activity level from the group average within ±15% to 30%; the frequency of mounting behaviors during non-estrus periods not exceeding 0.5 times the group average; and the frequency of aggressive behavior ranging from 0.5 to 1.5 times the group average.
- Poor cattle behavior is characterized by: deviations in feeding and drinking behaviors from the group baseline exceeding ±25%; standing time exceeding 1.5 times the group average, or nighttime lying time accounting for less than 45%; deviations in activity level from the group average exceeding ±30%; the frequency of mounting behaviors exceeding 1.5 times the group average, or frequent occurrence during non-estrus periods; and the frequency of aggressive behavior exceeding 1.5 times the group average, or each incident lasting more than 5 min.

#### 2.1.2 Environmental data

To ensure the health and welfare of cattle, environmental data, including temperature, humidity, CO_2_ levels, and light intensity, are monitored to optimize living conditions, prevent health issues caused by extreme conditions such as heat stress or cold, reduce disease incidence, and improve comfort and productivity. This ensures that the cattle are kept in a suitable environment, thus enhancing their overall welfare. Based on the standards “HJ 568–2010 Livestock and Poultry Farming Environmental Evaluation Guidelines” (22) and “NY/T 2363–2013 Dairy Cattle Heat Stress Evaluation Technical Guidelines” (23), the evaluation criteria for environmental data, including temperature, humidity, light intensity, and CO_2_ concentration, are shown in Table 2.

**Table 2:** Evaluation criteria of environmental indicators.

No.	Index/unit	Excellent	Medium	Low
1	Temperature/°C	15~22	5~15, 22~26	<5, >26
2	Humidity/%RH	55~75	40~55, 75~85	0~40, 85~100
3	Light intensity/lx	200~300	50~200	0~50
4	CO_2_ concentration/ppm	800~1,500	1,500~2,000	>2,000

**Figure 1:**
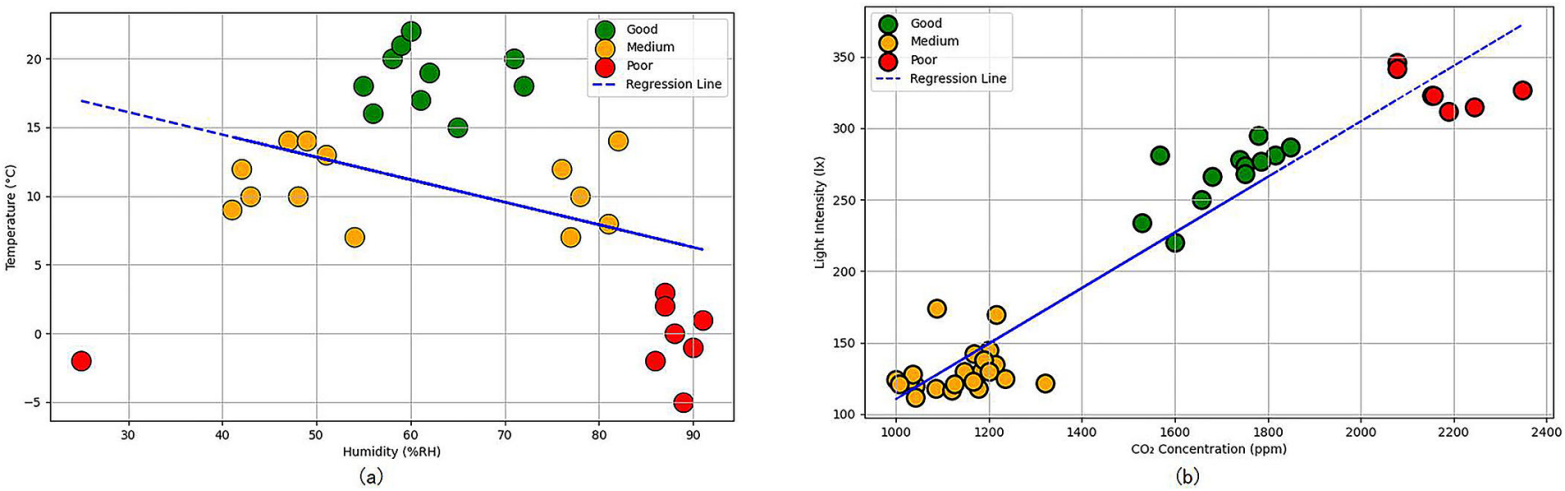
Environmental data regression analysis plot: **(a)** humidity as the x-axis and temperature as the y-axis; **(b)** carbon dioxide concentration as the x-axis and light intensity as the y-axis.

Based on the measurement results of the farm barn environmental data, the correlations between temperature and humidity, light intensity and carbon dioxide concentration, and cattle welfare were analyzed. As shown in Figure 1a, humidity was set as the horizontal axis and temperature as the vertical axis, with welfare levels as the basis for regression analysis. The results indicated a significant correlation between the combination of temperature and humidity and cattle welfare. Similarly, as shown in Figure 1b, carbon dioxide concentration was set as the horizontal axis and light intensity as the vertical axis. Regression analysis based on welfare levels revealed a clear relationship between the interaction of carbon dioxide concentration and light intensity and cattle welfare.

#### 2.1.3 Feeding data

Farm feeding data, including the amount of feed, nutritional components, and feeding patterns, are monitored to help optimize cattle health, productivity, and behavioral performance. This approach prevents health issues, nutritional imbalances, or behavioral abnormalities caused by insufficient or excessive feeding, thereby providing personalized management to enhance overall cattle welfare. Based on the standards “NY/T 3049-2016 Technical Regulations for Dairy Cattle Total Mixed Ration Production” (24), feeding data such as dry matter (DM), acid detergent fiber (ADF), neutral detergent fiber (NDF), crude protein (CP), calcium (CA), and phosphorus (P) are obtained, with the proportions of nutrients extracted from the feed being used as feeding data. The evaluation criteria for these indicators are shown in Table 3.

**Table 3:** Feeding index evaluation criteria.

No.	Index/unit	Excellent	Medium	Low
1	Dry matter DM%	50~75	75~85	0~50, 85~100
2	Acid detergent fiber ADF%	17~21	21~27	27~40
3	Neutral detergent fiber NDF%	25~33	34~47	47~60
4	Crude protein CP%	12~18	18~21	21~27
5	Calcium CA%	0.6~1.2	1.2~2	0~0.6
6	Phosphorus P%	0.3~0.7	0.7~1	0~0.3

**Figure 2:**
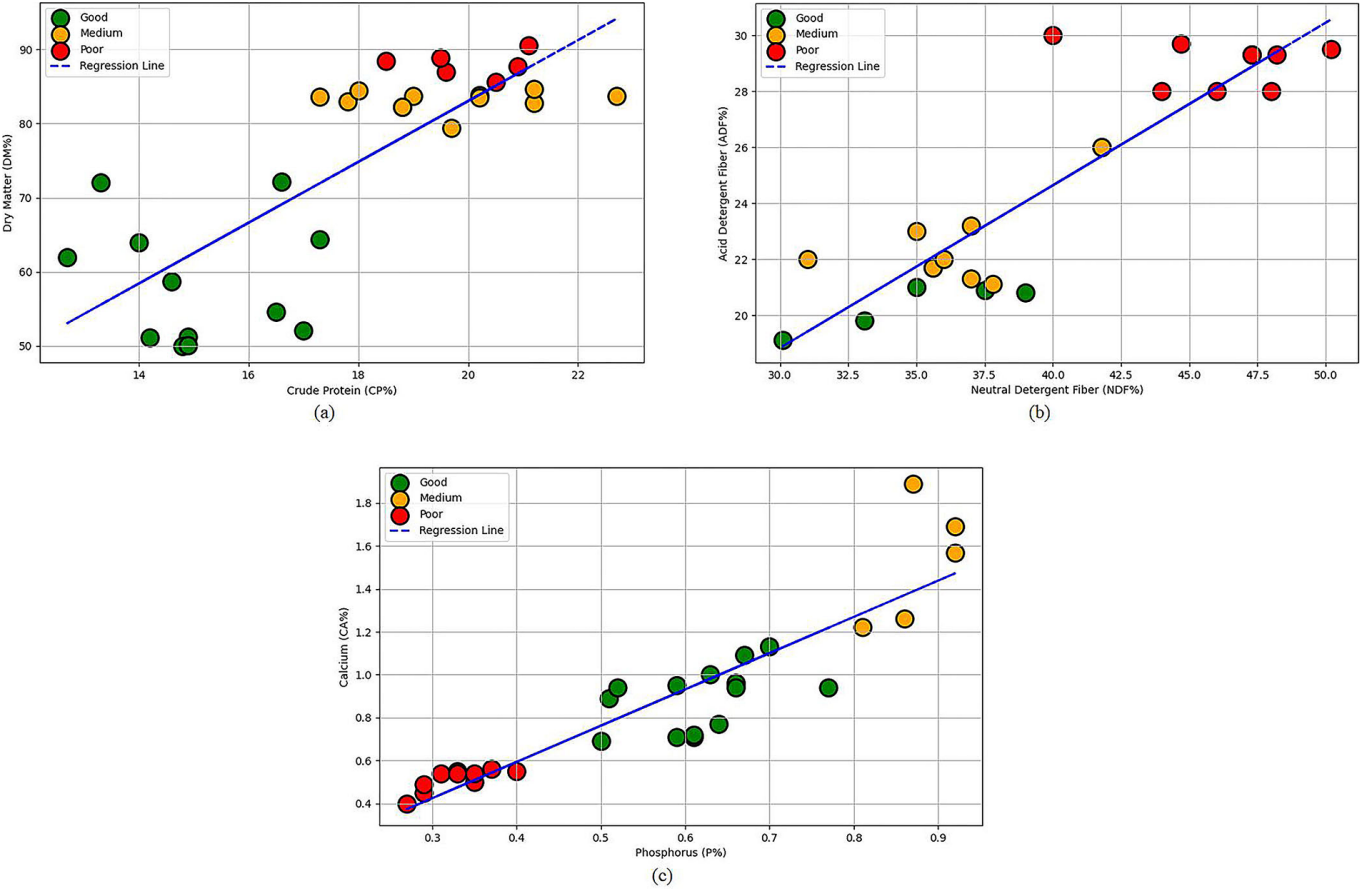
Feeding data regression analysis plot: **(a)** crude protein content as the x-axis and dry matter content as the y-axis; **(b)** neutral detergent fiber content as the x-axis and acid detergent fiber content as the y-axis; **(c)** phosphorus content as the x-axis and calcium content as the y-axis.

By analyzing the feeding data obtained from the farm feeding database and dairy cattle feed formulas (25), the relationships between dry matter content, acid detergent fiber (ADF) content, neutral detergent fiber (NDF) content, crude protein content, calcium content, and phosphorus content with cattle welfare were explored. As shown in Figure 2a, crude protein content was set as the horizontal axis and dry matter content as the vertical axis, with welfare levels incorporated into the regression analysis. The results indicated that crude protein and dry matter content significantly influence welfare levels. Similarly, as shown in Figure 2b, neutral detergent fiber content was set as the horizontal axis and acid detergent fiber content as the vertical axis, with regression analysis based on welfare levels showing a close relationship between these two fiber contents and cattle welfare. Additionally, as shown in Figure 2c, phosphorus content was set as the horizontal axis and calcium content as the vertical axis. The analysis further revealed a clear connection between the variations in phosphorus and calcium content and welfare levels.

### 2.2 BP neural network

**Figure 3:**
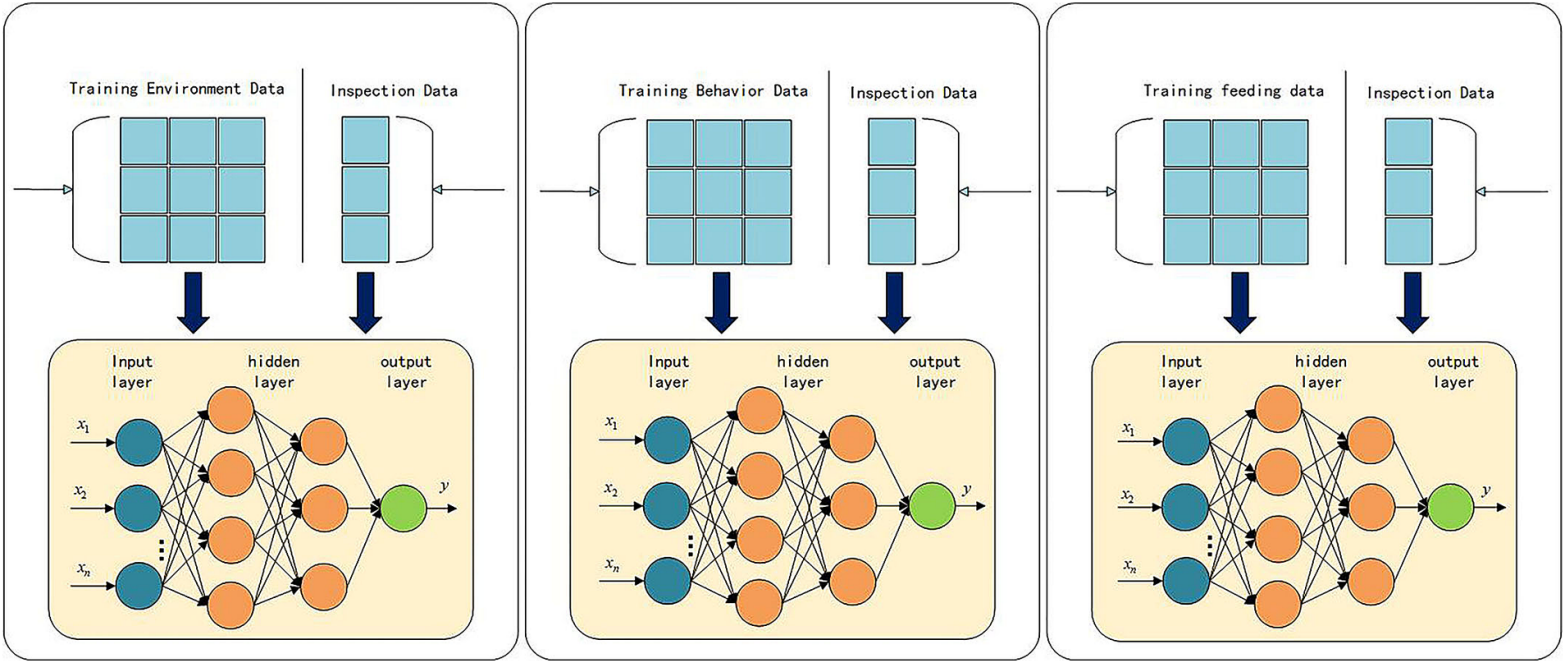
BP neural network structure diagram.

In the fuzzy neural network system, three parallel BP neural network structures are used, each responsible for classifying the three types of data—feeding, environment, and behavior—into results that can be used by the fuzzy inference system. Through the hierarchical structure of the BP neural network and the backpropagation algorithm, the system learns patterns from complex data and generates accurate preliminary classification results, providing a foundation for fuzzy inference. The network structure diagram is shown in Figure 3.

The main function of the BP neural network is to extract features from multi-source heterogeneous data and generate the input levels required for the fuzzy inference system. Specifically, the BP neural network processes feeding data, environmental data, and behavioral data, classifying them into fuzzy levels (e.g., low, medium, high). These outputs provide the foundation for subsequent fuzzification and fuzzy inference. The environmental, feeding, and behavioral indicators from the farm serve as the inputs to the BP neural network, with the outputs being environmental levels, feeding levels, and behavioral levels, respectively. The various indicators and their corresponding meanings are shown in Table 4.

**Table 4:** Indicators and their alphabetical meaning tables.

No.	Category	Indicators	Input	Letters indicate	Unit
1	Environmental indicators	Temperature	X_1_	T	°C
2		Humidity	X_2_	H	%RH
3		Light intensity	X_3_	Lux	lx
4		CO_2_ concentration	X_4_	CO_2_	ppm
5	Feeding indicators	Dry matter	X_5_	DM	%DM
6		Crude protein	X_6_	CP	%CP
7		Acid detergent fiber	X_7_	ADF	%ADF
8		Neutral detergent fiber	X_8_	NDF	%NDF
9		Calcium	X_9_	CA	%CA
10		Phosphorus	X_10_	P	%P
11	Behavior indicators	Behavior level	X_11_	Behavior	--

Three sets of parallel BP neural networks are modeled for environmental, feeding and behavioral data respectively, extracting features and independently outputting their welfare rating scores. The first group of input layer data $X=\left[x_{1},x_{2},\cdots ,x_{4}\right]$ input vector is environmental data; the second group of input layer data $X^{\prime }=\left[x_{5},x_{6},\cdots ,x_{10}\right]$ input vector is feeding data; the third group of input layer data $X^{\prime \prime }=\left[x_{11}\right]$ input vector is behavior data. The hidden layer data is h, the output layer data is y, the input layer to the hidden layer parameters are *ω*, b_1_, the hidden layer to the output layer parameters are v, b_2_, and the activation function is sigmoid represented by g_1_, g_2_.

Because the network is a three-group parallel BP neural network, the value range of each index is different. If it is not normalized, the index with larger eigenvalues will dominate the model training, resulting in unfair feature weight distribution. Therefore, in order to eliminate the difference of feature scale, the data distribution is concentrated in a unified range, such as [0,1]. The normalization formula is provided in Equation (5):

(5) $$x=\frac{x-x_{\text{min}}}{x_{\text{max}}-x_{\text{min}}}$$

In the formula, is the normalized eigenvalue, $x_{\text{min}}$ is the minimum value in all samples, and $x_{\text{max}}$ is the maximum value in all samples.

The BP neural network algorithm is described by the formulas in Equations (6) to (9):

(6) $$net_{1}=\omega^{T}x+b_{1},h=g_{1}\left(net_{1}\right)$$

(7) $$net_{2}=v^{T}h+b_{2},y=g_{2}\left(net_{2}\right)$$

(8) $$\begin{array}{l} y=g_{2}\left(net_{2}\right)=g_{2}\left(v^{T}g_{1}\left(net_{1}\right)+b_{2}\right)\\ =g_{2}\left(v^{T}g_{1}\left(\omega^{T}x+b_{1}\right)+b_{2}\right) \end{array}$$

(9) $$E\left(\theta \right)=\frac{1}{n}\overset{n}{\underset{i=1}{\sum }}{\left(y_{i}-{\overset{\wedge }{y}}_{i}\right)}^{2}$$

Here, $y_{i}$ is the true value, $\overset{\wedge }{y_{i}}$ is the predicted value, and n is the dimension of the output unit.

### 2.3 Fuzzy logic decision model

**Figure 4:**
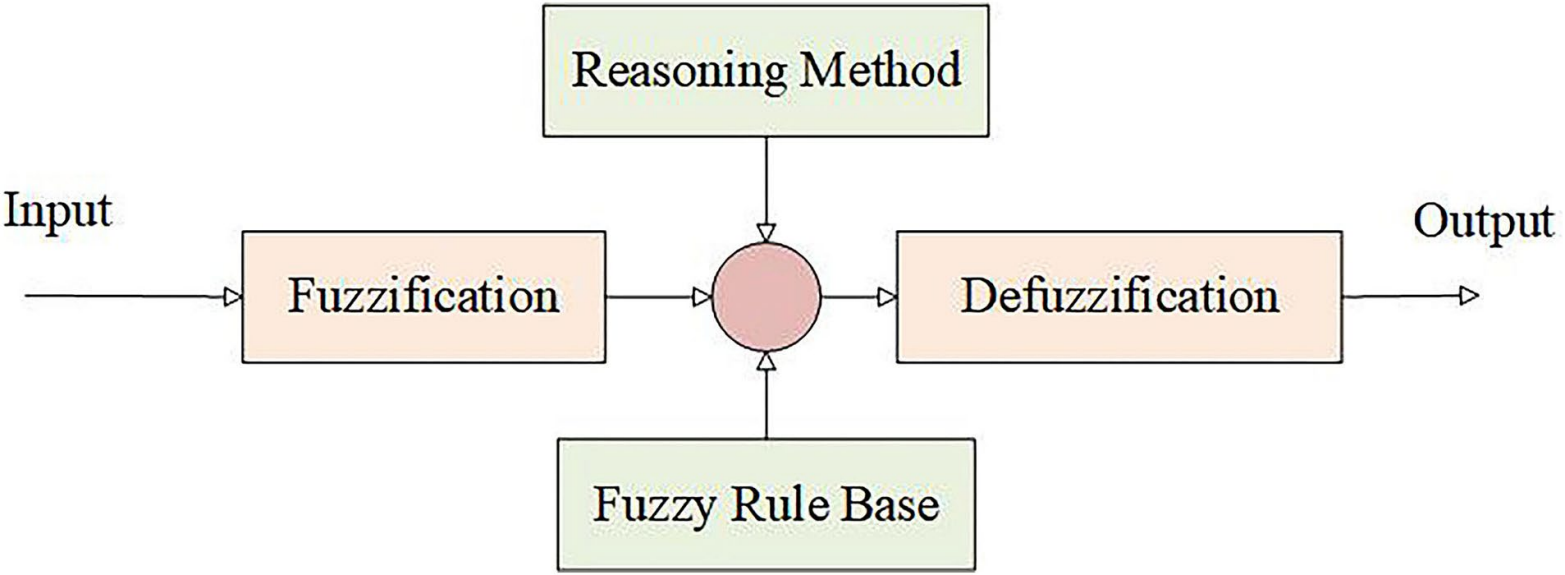
Fuzzy logic architecture diagram.

Fuzzy logic is the core component of the fuzzy neural network, used to simulate the fuzzy reasoning capabilities of human thinking. It maps continuous input data into fuzzy sets using membership functions, fuzzy rules, and reasoning mechanisms, and produces decision outputs based on the defined fuzzy rule table. The fuzzy logic architecture consists of four main parts: fuzzification, inference methods, fuzzy rules, and defuzzification, as shown in Figure 4.

#### 2.3.1 Input fuzzification

First, the environmental levels, feeding levels, and behavioral levels of the farm are defined as input variables, while the overall welfare level is defined as the output variable. The traditional welfare evaluation system uses triangular membership functions to represent the fuzzy characteristics of assessment indicators such as environment, feeding, and behavior. Although triangular membership functions are simple to compute, they have some limitations in practical applications. First, the function exhibits discontinuities at the vertex and endpoints, which do not align with the continuous nature of indicator changes in the actual evaluation process. Second, the use of linear changes to describe membership degrees fails to accurately reflect the nonlinear characteristics in real-world assessments. Finally, in the boundary regions of the evaluation indicators, the triangular membership function has limited expressive power, which can lead to distorted evaluation results.

To overcome the limitations mentioned above, this study introduces the Gaussian membership function to optimize system performance. The function is smooth and continuous across the entire domain, avoiding the discontinuities at critical points and effectively reflecting the gradual variation in evaluation indicators. Additionally, the Gaussian membership function provides a natural transition interval between different levels, with fuzziness gradually decreasing as the distance from the center value increases. This results in smoother boundary transitions and enhances the system’s robustness. For any two adjacent fuzzy sets, the membership degree variation in the overlap region follows a normal distribution, which better aligns with the fuzzy cognitive rules in the actual evaluation process. The mathematical expression of the Gaussian membership function is provided in Equation (10).

(10) $$\mu \left(x\right)=e^{-\frac{{\left(x-c\right)}^{2}}{2\sigma^{2}}}$$

Here, c is the center value of the Gaussian function, which represents the typical value of the fuzzy set; σ is the standard deviation, which is used to control the spread width of the function and determine the ambiguity degree of the fuzzy set.

**Figure 5:**
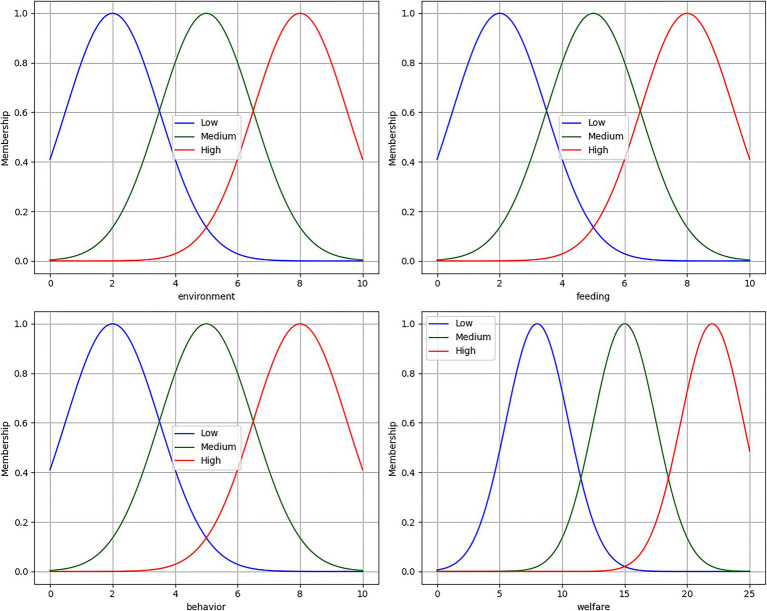
Membership function diagram.

After introducing the Gaussian membership function, the input and output values are transformed into fuzzy sets for easier inference in the subsequent rule base. The feature value table is shown in Table 5, and the membership functions for input and output are illustrated in Figure 5. The environmental level input is defined as “environment,” the feeding level input is defined as “feed,” the behavioral level is defined as “behavior,” and the overall welfare level output is defined as “welfare.” The specific fuzzy set divisions are as follows:

**Table 5:** Corresponding eigenvalue table of membership function.

No.	Definition	Eigenvalue	Low	Medium	High
1	Environment	Level	2	5	8
2	Feed	Level	2	5	8
3	Behavior	Level	2	5	8
4	Welfare	Level	8	15	22

Input Fuzzy Sets (membership degree range: [0,10])

- Environmental Level: {Low, Medium, High}
- Feeding Level: {Low, Medium, High}
- Behavior Level: {Low, Medium, High}

Common Parameters for Input Variables:

- Membership Function: Gaussian
- Standard Deviation: 1.5

Output Fuzzy Set:

- Comprehensive Welfare Level: {Low, Medium, High}
- Membership Degree Range: [0,25]
- Standard Deviation: 2.5

#### 2.3.2 Fuzzy rule formulation

By incorporating expert experience, fuzzy inference system rules are defined, mapping the fuzzy values of input variables to the fuzzy values of output variables through logical relationships. The specific fuzzy rules are shown in Table 6. Based on these fuzzy rules, the classical Mamdani fuzzy inference method is used for inference calculations. Through steps such as fuzzification, rule activation and aggregation, and defuzzification, the fuzzy sets of input variables are transformed into crisp values for the output variables, providing a scientific basis and reliable support for the system’s decision-making and evaluation.

**Table 6:** Fuzzy regulation table.

No.	Input			Output
	Environmental level	Feeding level	Behavior level	Welfare level
1	Low/Medium	Low	Low	Low
2	Low	Low/Medium	Low	Low
3	Low	Low	Low/Medium	Low
4	Low/Medium	Medium	Medium	Medium
5	Medium	Low/Medium	Medium	Medium
6	Medium	Medium	Low/Medium	Medium
7	Low	Medium/High	High/Medium	Medium
8	High	Low	Low	Medium
9	Low	High	Low	Medium
10	Low	Low	High	Medium
11	Medium	Low/High	High/Low	Medium
12	High	Low/Medium	Medium/Low	Medium
13	High	High	Low/Medium	High
14	High	Low/Medium	High	High
15	Low/Medium	High	High	High
16	Medium/High	Medium/High	High	High
17	Medium/High	High	Medium/High	High
18	High	Medium/High	Medium/High	High

This study designs a differentiated weight allocation mechanism based on the importance of evaluation rules to enhance the system’s ability to recognize key states, making the evaluation results more aligned with actual management needs. The mechanism adjusts the influence of different rule combinations in the decision-making process by assigning different weights. The weight distribution range is set between [1.0, 1.5], and the weight calculation formula is provided in Equation (11):


(11) $$W_{\mathrm{base}}=1+\alpha \cdot I_{\mathrm{consistency}}+\beta \cdot I_{\mathrm{importance}}+\gamma \cdot I_{\mathrm{correlation}}$$

Here, $W_{\mathrm{base}}$ is the basic weight value, α , β , and γ are the adjustment coefficients, which represent the influence degree of consistency, importance and correlation respectively, $I_{\mathrm{consistency}}$ represents the influence factor of index consistency, $I_{\mathrm{importance}}$ is the influence factor of rule importance, and $I_{\mathrm{correlation}}$ is the influence factor of index correlation.

The calculation formula for the indicator consistency influence factor is provided in Equation (12):


(12) $$I_{\mathrm{consistency}}=1-\frac{\left|\max\left(\mu_{i}\right)-\min\left(\mu_{i}\right)\right|}{N}$$

Among them, $\mu_{i}$ represents the membership value of each index, and N is the number of evaluation indexes. When the evaluation results of each index tend to be consistent, the factor value is larger, and the overall weight of the rule is improved.

The formula for calculating the rule importance factor is presented in Equation (13):


(13) $$I_{\mathrm{impor}\tan ce}=\frac{\sum w_{i}\times v_{i}}{\sum w_{i}}$$

$w_{i}$ is the preset weight of each evaluation index, and $v_{i}$ is the corresponding evaluation value. The calculation method takes into account the relative importance of different indicators in the overall assessment.

The formula for calculating the indicator-related impact factor is shown in Equation (14):


(14) $$I_{\mathrm{correlation}}=1+\frac{\rho \times \left(\sum \left(x_{i}-\bar{x}\right)\left(y_{i}-\bar{y}\right)\right)}{\left(\sigma_{x}\times \sigma_{y}\right)}$$

In the equation, ρ is the correlation coefficient adjustment parameter, $\bar{x}$ and $\bar{y}$ are the mean values of the indicators, $\sigma_{x}$ and $\sigma_{y}$ are the standard deviations. This factor reflects the degree of interaction between different indicators.

### 2.4 Fuzzy neural network fusion algorithm

#### 2.4.1 Preliminary processing and feature extraction of multi-source data

The multi-source data (environment, feeding, behavior) is organized and cleaned to ensure data integrity and consistency, followed by normalization to address the differences in measurement scales. After normalization, the data is input into three parallel BP neural networks, where the networks’ nonlinear mapping ability outputs the environmental level, feeding level, and behavioral level. This process achieves preliminary feature extraction and structured representation of the multi-source data, providing the foundational input for subsequent fuzzy logic decision-making.

#### 2.4.2 Fuzzy logic decision making

Based on the environmental level, feeding level, and behavioral level, fuzzyfication is performed using a fuzzy logic system. The fuzzification process converts crisp numerical levels into membership degrees, mapping the data to fuzzy sets {Low, Medium, High} using the Gaussian membership function. A rule base, defined by expert experience and real-world application scenarios, establishes the logical relationships between the environment, feeding, behavior, and overall welfare levels. Inference is conducted through fuzzy rules, mapping the fuzzy values of input variables to the fuzzy values of output variables, ultimately resulting in the fuzzy set for the overall welfare level.

#### 2.4.3 Comprehensive evaluation and welfare level output

Based on fuzzy logic decision-making, the fuzzy output sets from the activated rules are aggregated to obtain the fuzzy output result for the overall welfare level. To achieve a clear evaluation, the fuzzy set is transformed into a specific crisp value through defuzzification, resulting in the final overall welfare score. Additionally, by dividing the score into grade intervals, this process not only quantifies the overall welfare status but also provides users with intuitive information about the level classification.

Building on this, the study introduces an adaptive scoring mechanism aimed at optimizing the evaluation process and improving the accuracy and applicability of the results. This mechanism dynamically monitors the overall level of input indicators and automatically adjusts the boundary range of the final score to avoid biases that may arise from fuzzy inference, ensuring that the system’s output reflects the actual management level accurately and provides effective decision-making support for management improvements. By establishing an adaptive relationship between input indicators and scoring results, the system can more precisely differentiate between different management levels and provide evaluation results that are more aligned with reality.

An adaptive scoring adjustment mechanism based on multi-dimensional features:

The comprehensive mean calculation equation is presented in Equation (15):


(15) $$Avg_{\mathrm{input}}=\frac{w_{e}\times \mathrm{environment}+w_{f}\times \mathrm{feeding}+w_{b}\times \mathrm{behavior}}{w_{e}+w_{f}+w_{b}}$$

Among them, $w_{e}$ , $w_{f}$ and $w_{b}$ are the weight coefficients of environmental, feeding and behavioral indicators, respectively.

High segment adaptive adjustment is given in Equation (16): when $Avg_{\mathrm{input}}\geq 8.5$ :


(16) $$\mathrm{welfar}e_{\mathrm{score}}=\max\left(\mathrm{origina}l_{\mathrm{score}},20+\lambda \left(Avg_{\mathrm{input}}-8.5\right)\right)$$

Among them, λ is the high-segment adjustment coefficient.

Low segment adaptive adjustment is given in Equation (17): when $Avg_{\mathrm{input}}\leq 3$ :


(17) $$\mathrm{welfar}e_{\mathrm{score}}=\min\left(\mathrm{origina}l_{\mathrm{score}},20-\mu \left(3-Avg_{\mathrm{input}}\right)\right)$$

Here, μ is the low-segment adjustment coefficient.

### 2.5 Experimental platform and parameter settings

The experimental test platform is carried out in the Windows10 operating system environment. The CPU is Intel (R) Core (TM) i7-10700F, the main frequency is 2.90GHz, the memory is 64GB, and the graphics card is NVIDIA GeForce RTX 4070. The algorithm development platform is Visual Studio Code, the programming environment is Python 3.9, and the deep learning framework is Pytorch 1.10.0.

Set the batch-size parameter to 64, train 1,000 rounds, the learning rate is 0.01, the optimizer uses SGD, and the loss function is MSELoss.

## 3 Results

### 3.1 Model training

**Figure 6:**
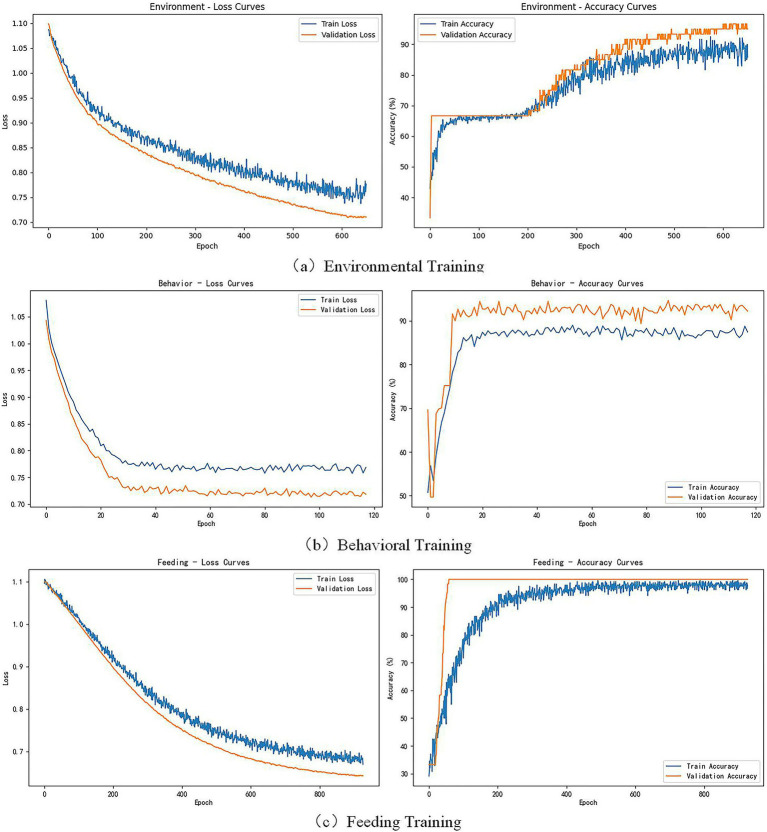
BP training result chart. **(a)** Environmental training. **(b)** Behavioral training. **(c)** Feeding training.

The BP neural network model constructed for the three dimensions of environment, feeding, and behavior first normalizes the three types of input data, mapping the feature values uniformly to the [0, 1] range to eliminate the impact of dimensionality. The dataset is divided into training and validation sets at an 8:2 ratio, with stratified sampling ensuring consistency in data distribution. In terms of model structure, the environment evaluation module uses a [64, 32] double hidden layer structure, the feeding evaluation uses a [128, 64] structure, and the behavior evaluation uses a [32, 16] structure. After training, the environment evaluation module achieved accuracy rates of 88.7% and 95.0% on the training and validation sets, respectively; the feeding evaluation module achieved 98.3% and 100%, respectively; and the behavior evaluation module achieved 85.7% and 93.6%. The model demonstrated strong generalization capability across all dimensions, with validation accuracies exceeding 90%. The training process and model performance evaluation results are shown in Figure 6.

### 3.2 Analysis of experimental results

**Figure 7:**
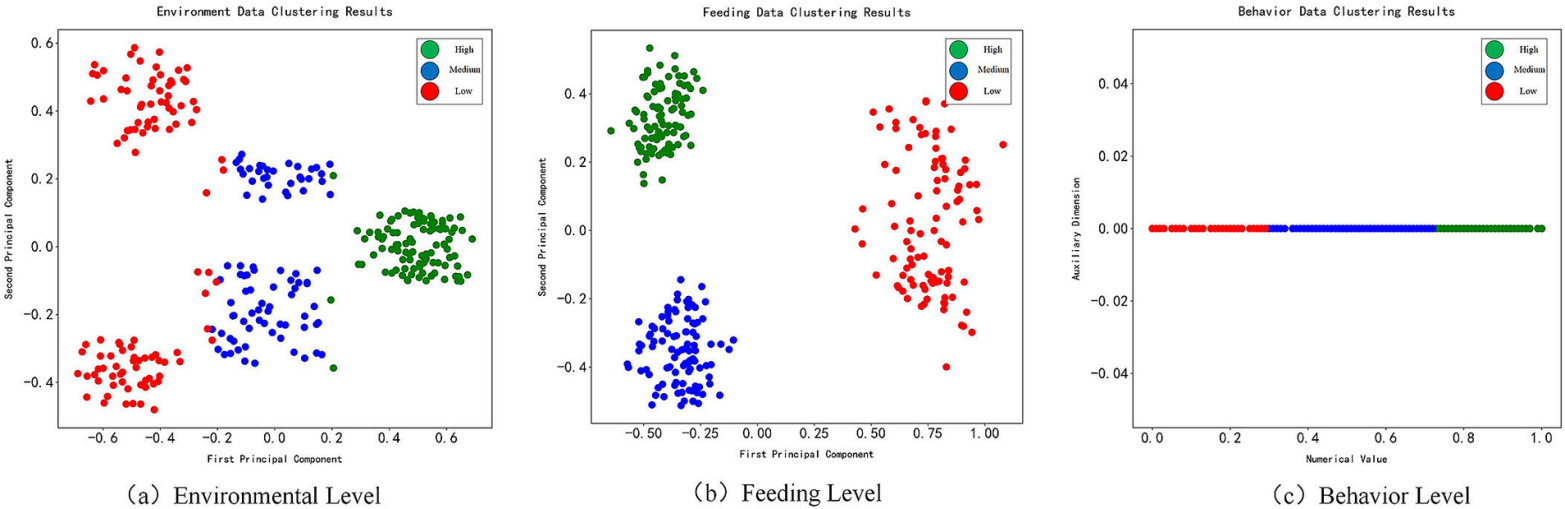
Fusion clustering distribution map. **(a)** Environmental level. **(b)** Behavioral level. **(c)** Feeding level.

The clustering analysis results of the three-dimensional data based on the BP neural network model are shown in Figure 7. The indicators from each dimension distinguish the welfare levels of the cattle effectively. The behavior data exhibit a clear linear separation in one-dimensional space, with the “Excellent,” “Good,” and “Poor” levels clearly distributed within the [0, 1] range, reflecting the continuity and reliability of the scoring criteria. After PCA dimensionality reduction of the environmental data, the “Excellent” samples concentrate on the right side, while the “Poor” samples are distributed across two clusters on the left, indicating that environmental factors have a multi-modal influence. The clustering of feeding data is the most effective, with the three levels significantly separated in two-dimensional space. In particular, the “Excellent” and “Good” samples are tightly clustered, while the “Poor” samples, though scattered, have clear boundaries, validating the model’s high accuracy and emphasizing the crucial role of feeding indicators in animal welfare evaluation.

**Figure 8:**
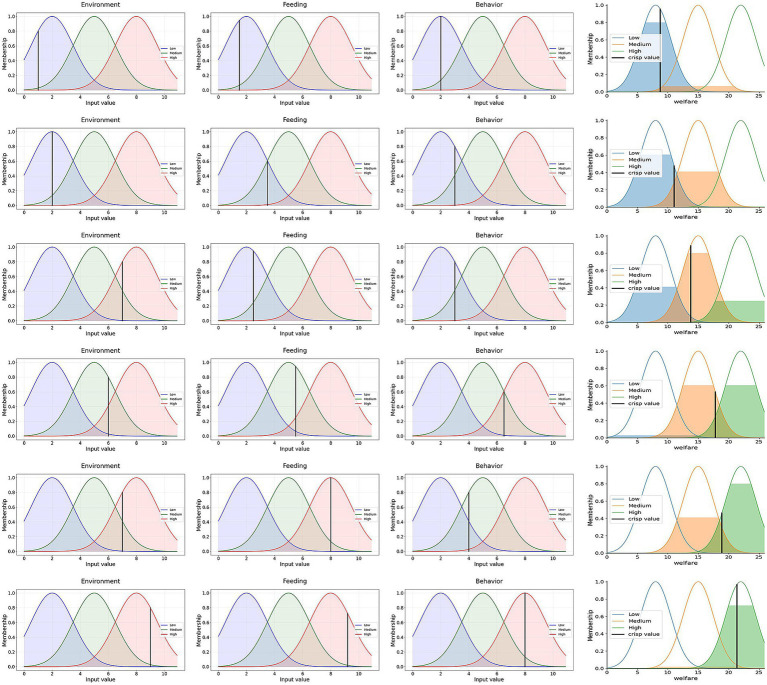
Predicted welfare level result chart.

To validate the effectiveness of the model, six sets of environmental, feeding, and behavioral data were selected for welfare level evaluation, and the corresponding membership function graphs were generated, as shown in Figure 8. By analyzing the shape and trends of the membership functions, the impact of different environmental conditions, feeding strategies, and animal behaviors on animal welfare can be intuitively observed. To verify the reliability of the model’s evaluation results, experienced managers with years of farming knowledge were invited to conduct manual welfare level assessments on the same six sets of data, based on the previously mentioned grading standards for environmental parameters, feeding management, and behavioral performance. After ensuring a consistent understanding of the evaluation criteria, the evaluators independently completed the assessments. The results, after consistency analysis, were compared with the model’s predictions, leading to the construction of the fuzzy logic decision experiment results table (see Table 7).

**Table 7:** The experimental results of fuzzy logic control.

No.	Input			Output		Control decision
	Environmental level	Feeding level	Behavior level	Prediction	Artificial evaluation	
1	1	1.5	2	8.8 = Low	Low	Optimize the pasture environment and feeding nutritional value
2	2	3.5	3	11.1 = Low	Low	
3	7	2.5	3	13.8 = Medium	Medium	
4	6	5.5	6.5	17.8 = Medium	Medium	
5	7	8	4	18.9 = High	High	Behavioral observation and management of cattle maintain
6	9	9.2	8	21.4 = High	High	

In cases where the predicted results are poor or moderate, corresponding environmental, feeding, and behavioral observation and management measures can be formulated based on the fuzzy logic decision table to optimize cattle welfare. When environmental and feeding conditions meet the standards, but behavioral performance is lacking, the focus should be on identifying the causes of behavioral abnormalities in the cattle. Measures such as adjusting group density and enrichment strategies should be implemented to improve behavior. On the other hand, when all indicators perform excellently, the existing feeding management strategies can be maintained to ensure the stability and improvement of cattle welfare. This multi-dimensional evaluation approach provides a more comprehensive reflection of cattle welfare, offering scientifically sound and rational optimization solutions for actual breeding management from various perspectives, including environment, nutrition, and behavior.

## 4 Discussion

Although this study collects behavioral data by selecting specific time periods and combining them with cattle activity characteristics, the current 10-min intermittent monitoring scheme still has certain limitations in fully characterizing cattle behavior due to the processing capabilities of edge devices. It should be noted that this system is primarily suitable for the following application scenarios: (1) barn environments with good monitoring locations; (2) cattle groups of 15–20 animals that can be captured in a single shot. The system may have limitations in the following scenarios: (1) environments with insufficient lighting; (2) scenarios with excessively high cattle density; (3) applications requiring the identification of complex behavior patterns. Due to the relatively slow movement of cattle, the complete cycle of some behavioral patterns may exceed 10 min, which may lead to fragmented collection of behavioral features. To address this issue, two optimization strategies are proposed: first, for individuals whose nutritional needs and behavior performance do not match (e.g., cattle with abnormal behavior but meeting feeding conditions), a multi-period cross-monitoring strategy is used. This involves adding 2–3 supplementary monitoring periods to the original monitoring period, with time-shifted sampling to obtain more complete behavior sequences; second, a graded monitoring mechanism is established, where individuals identified as abnormal during the initial monitoring undergo continuous tracking observations for 3 days, with one sample taken at different time periods each day to confirm the stability of their behavior patterns. Although these optimization strategies can partially compensate for the shortcomings of single short-term monitoring, challenges such as difficulty in data integration and increased manual verification workload still exist in practical applications. Therefore, during implementation, monitoring frequency and duration should be adjusted flexibly based on the actual management needs and resource conditions of the farm to balance monitoring effectiveness and operational feasibility. Additionally, this study lacks detailed differentiation of complex behaviors, such as distinguishing between standing and walking. Therefore, in the future, gait scoring and body condition scoring systems based on deep learning can be referenced to improve the accuracy and completeness of behavior classification (26). With the rapid development of deep learning technology, behavior recognition systems based on deep learning have been widely applied to livestock behavior monitoring and have made significant progress in overcoming the limitations of traditional computer vision methods (e.g., occlusion, lighting changes, and complex scenes) (27, 28). Currently, there are three main technical approaches to cattle behavior monitoring: wearable sensor-based solutions (29), computer vision-based solutions (30), and multi-sensor fusion solutions (31). Compared with wearable sensor solutions, the visual solution used in this study does not require cattle to wear devices, significantly reducing application costs and animal stress responses. While the intermittent monitoring strategy proposed by this system has certain limitations in fully characterizing behavior compared to existing continuous visual monitoring solutions, it maintains basic monitoring effectiveness while significantly reducing system resource consumption by optimizing monitoring periods and sampling strategies. Combining the previously proposed multi-period cross-monitoring and graded monitoring mechanisms can further address the limitations of intermittent monitoring and achieve a good balance between resource efficiency and monitoring effectiveness.

The evaluation method in this study primarily targets barn environments. Considering the limited computational resources of edge devices, the system adopts a lightweight data processing scheme that effectively reduces the edge computing load while ensuring evaluation accuracy. Model pruning and quantization techniques can further optimize computational efficiency, but they may sacrifice detection accuracy (32). Therefore, performance can be improved by enhancing the detection module or utilizing hardware acceleration on edge devices. Alternatively, a distributed computing solution involving collaboration between edge devices and local servers can be explored. The evaluation framework in this study has good scalability, and by adjusting the evaluation indicators and data collection strategies, it can be adapted to different types of livestock environments, such as dairy farms, beef cattle farms, and sheep farms. Additionally, the system can be extended to applications outside barn environments. For example, based on research by Laschinger et al. (33), by adjusting evaluation indicators (such as weight transfer and abnormal standing posture) and sampling strategies, cattle behavior monitoring can be implemented in different types of milking parlors, such as fishbone, parallel, and tandem parlors. The system not only supports the integration of various data collection devices but also allows flexible adjustment of evaluation criteria based on the physiological characteristics and behavioral patterns of different livestock species, offering a universally valuable technical solution for intelligent management in the livestock industry.

Based on the evaluation method and experimental results of this study, future research and applications can be developed in the following areas: First, at the system technology level, the accuracy of behavior recognition can be improved by introducing advanced algorithms such as deep learning. Additionally, by integrating physiological sensor data (e.g., body temperature, heart rate) and environmental data (e.g., temperature, humidity, ammonia, and methane concentrations), the evaluation dimensions can be expanded to build a more comprehensive welfare assessment system (34, 35). Moreover, devices such as RFID tags on farms and inspection robots can provide valuable data support for deep learning models (36). Second, in terms of application scenarios, plans include deeply integrating this evaluation system with existing production management systems on farms, enabling the linkage of welfare assessment results with daily feeding management data to support precision farming decisions. For example, based on welfare evaluation results, feeding plans and environmental parameters can be automatically adjusted, or individualized management measures can be implemented. Additionally, efforts will be made to establish a regional cattle welfare evaluation database. Through data sharing and comparative analysis, this database will help farms develop more targeted welfare improvement programs and provide scientific evidence for industry standards. Finally, in terms of promotion and application, the system’s suitability in different-scale and different-type farms will be validated through demonstration projects. Based on these results, supporting technical training materials and application guides will be developed to promote the widespread adoption of the evaluation system and elevate the overall welfare standards in livestock farming.

## 5 Conclusion

This study proposes an adaptive fuzzy logic-based cattle welfare evaluation method integrated with multi-modal data fusion. The method establishes a comprehensive evaluation system across three dimensions: environment, feeding, and behavior. By designing a quantitative scoring mechanism for behavioral duration and individual group differences, the method effectively represents behavior data. In model construction, three parallel BP neural networks are employed to extract features and perform preliminary classification of environmental, feeding, and behavioral data. The traditional triangular membership function is replaced with a Gaussian membership function in the fuzzy logic decision-making process, enhancing the system’s robustness and evaluation accuracy. Additionally, a differentiated weight allocation mechanism is designed to improve the system’s ability to recognize critical states, and a dynamic adaptive scoring mechanism is developed to automatically adjust score boundaries based on the overall level of input indicators. Experimental results demonstrate that the method shows excellent performance and good generalization ability across all evaluation dimensions: the environment evaluation module achieves accuracy rates of 88.7% and 95.0% for the training and validation sets, respectively; the feeding evaluation module achieves 98.3% and 100%, respectively; and the behavior evaluation module achieves 85.7% and 93.6%. The validation accuracy for all dimensions exceeds 90%, with high consistency with manual evaluation results. Based on the evaluation results, farm managers can implement targeted environmental control, feeding optimization, and behavior management measures, providing effective technical support for the intelligent, scientific, and integrated management of modern farms.

## Data Availability

The raw data supporting the conclusions of this article will be made available by the authors, without undue reservation.
