# Supplementary material for: Cattle welfare assessment based on adaptive fuzzy logic and multimodal data fusion
Source: Front Vet Sci. 2025 Apr 9;12:1568715. doi: 10.3389/fvets.2025.1568715 (PMC12016884; doi:10.3389/fvets.2025.1568715)

Supplementary Material

# Supplementary Tables

**Supplementary Table 1.** **Behavior data.**

To present behavioral data classification and characteristics more intuitively, this study refined behavioral categorization based on the "Five Freedoms" principle, as shown in Table 1, detailing specific classifications, characteristic descriptions, and perception methods.

| No. | Behavior Classification | Specific Behavior | Behavior  Characteristics | Perception Methods |
| --- | --- | --- | --- | --- |
| 1 | Physiological behavior | feeding | Duration, frequency and interval of feeding. Under normal circumstances, feeding time is within a certain range, and eating too short or too long may reflect feed quality problems or health problems. | visual perception |
| 2 | Physiological behavior | drinking | The frequency, duration and amount of drinking water. Abnormal drinking behavior, such as too high or too low frequency of drinking water, may indicate abnormal ambient temperature or cattle health problems (such as fever, stress). | visual perception |
| 3 | Physiological behavior | standing | Time, frequency and duration of standing. Long-term standing or frequent standing may mean uncomfortable environment, pain or unsuitable padding. | visual perception |
| 4 | Physiological behavior | lying | The length of lying time, posture and number of ups and downs. Adequate lying time is very important for the rumination and comfort of cattle. Too much or too little lying may indicate health problems (such as leg pain and environmental discomfort). | visual perception |
| 5 | Sports behavior | walking | The number of walks, steps, distance. Normal walking can promote health, but insufficient walking may indicate laziness or leg problems, and excessive walking may be due to anxiety or environmental discomfort. | visual perception |
| 6 | Social behavior | fighting | Including the frequency and intensity of the fight between cattle. Frequent fighting may be a manifestation of social pressure or lack of space. | visual perception |
| 7 | Social behavior | climbing | This behavior is generally related to estrus. If the non-estrous climbing behavior is frequent, it may indicate group management problems or excessive pressure on cattle. | visual perception |

# Supplementary Figures

**Supplementary Figure 8.** Predicted welfare level result chart. In order to verify the validity of the model, six groups of environmental, feeding and behavioral data were selected to evaluate the welfare level, and the corresponding membership function diagram was output.


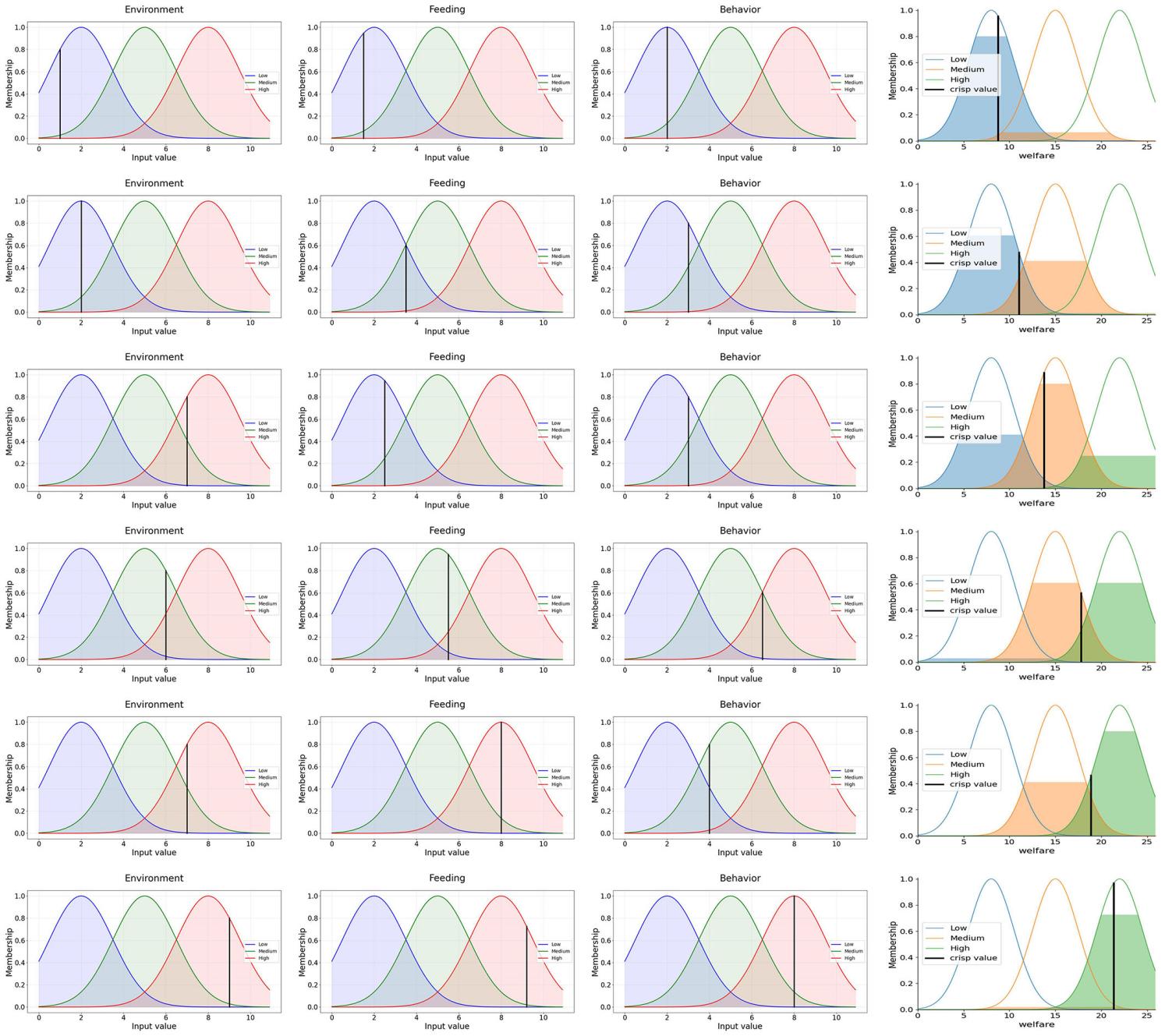

Supplement: Supplementary file 1 [file Table_1.DOCX]
